# Neural Responses During Emotion Transitions and Emotion Regulation

**DOI:** 10.3389/fpsyg.2021.666284

**Published:** 2021-08-18

**Authors:** Yu Hao, Lin Yao, Gary W. Evans

**Affiliations:** ^1^Department of Design and Environmental Anlaysis, Cornell University, Ithaca, NY, United States; ^2^Frontiers Science Center for Brain & Brain-Machine Integration, Zhejiang University, Hangzhou, China; ^3^College of Computer Science, Zhejiang University, Hangzhou, China; ^4^Department of Human Development, Cornell University, Ithaca, NY, United States

**Keywords:** emotion transitions, temporal context, context dependency, functional connectivity, frontal connectivity, cognitive reappraisal, flexibility, adaptation

## Abstract

Why are some people more susceptible to interference from previous emotional stimuli? Neural mechanisms underlying emotion regulation are typically studied with one-off positive or negative stimuli. Less is known about how they operate during dynamic emotional experiences, which more closely resemble how emotions occur in real life. Therefore, we investigated the interaction among temporal context, stimulus content, and regulatory strategy. Image sequences included either neutral to negative emotion or negative to neutral emotion. Participants were instructed to either passively watch the emotional stimuli or apply cognitive reappraisal during the image sequences presentation. Participants also reported their habitual use of cognitive reappraisal in their daily lives on a standard scale. We measured functional connectivity (FC) with electroencephalography (EEG) source localization. A three-way interaction suggested that, in addition to momentary emotional content and regulatory effort, the temporal context of stimuli impacts the FC between the ventromedial prefrontal cortex (vmPFC) and the ventral anterior cingulate cortex (ACC) in both alpha and beta frequency bands. In the reappraisal condition—but not the passive watch conditions—, individual differences in habitual reappraisal were manifested in the FC of vmPFC-ACC in alpha band. Emotion transitions may be more demanding because prefrontal-posterior FC in the beta band decreased during emotion transitions regardless of emotional content or regulation efforts. Flexible emotion regulation enables the recruiting of neural activities in response to the content of dynamic, ever-changing experiences encountered in daily life. Studying brain responses to dynamic emotional stimuli may shed light on individual differences in adaptation and psychological health. It also provides a more ecologically valid assessment of emotion regulation.

## Introduction

Emotions are important in influencing how we think and behave. However, emotional experiences in real life rarely occur in a vacuum. We are affected not only by the context in which the experience occurs (Pastor et al., 2008; Fujimura et al., 2013; Czekóová et al., 2015), but as agentive creatures, we may attempt to change or regulate what we are currently feeling (Gross, 1998). Therefore, emotion processing incorporates the interaction of situation, regulation, and person factors (Aldao, 2013; Koole and Veenstra, 2015; Doré et al., 2016). The dynamic features of emotion are critical to emotion regulation flexibility and adaptation to environmental change, and may be linked to psychopathology (Aldao et al., 2015; Hollenstein, 2015; Heller and Casey, 2016). To date, most research on the neural substrates of temporal dynamics of emotions has focused on reactions to stimuli within a single emotional category, such as negative content (e.g., Gianotti et al., 2008; Thiruchselvam et al., 2011; Dan-Glauser and Gross, 2015). This also means that the examination of emotion regulation has largely been studied apart from emotion dynamics.

As an important emotion regulation strategy, cognitive reappraisal involves attempts to alter the meaning of stimuli to diminish negative affect (Gross, 1998; Ochsner et al., 2012). An example of reappraisal includes thinking an aversive situation will eventually become better. Cognitive reappraisal reflects a form of cognitive control based in the prefrontal cortex (PFC), which regulates activation in the ventral emotion generative regions (Wager et al., 2008; Ochsner et al., 2012). In most studies investigating the neural bases of cognitive reappraisal, the stimuli were one-offs, and participants were instructed whether or not to reappraise a given stimulus. More extended temporal dynamics, especially during emotion regulation efforts when stimuli shift, have received little attention. Thus, the first goal of the present study is to investigate whether neural responses differ depending on whether an emotion is preceded by another differing emotion, and whether the dynamic emotion experiences vary with the use of cognitive reappraisal.

Temporal context influences emotional perception and neural responses (Pastor et al., 2008; Fujimura et al., 2013; Czekóová et al., 2015). For example, the perception of neutral images is more negative if they are preceded by negative images or descriptions than when they are preceded by non-negative ones (Pastor et al., 2008; Tambini et al., 2017; Grupe et al., 2018). Moreover, emotion regulation might have enduring behavioral and neural effects (Foti and Hajcak, 2008; MacNamara et al., 2011). Here we examined whether the manipulation of emotion stimuli in the temporal context matters for emotion regulation. Emotion transitions were elicited through two different contexts: by presenting an image sequence with increasing or decreasing negative affect. We also instructed participants to either passively watch the image sequence or invoke cognitive reappraisal. Such a design allowed us to assess within-subject responsivity to emotional content, context, and regulation strategy. This approach also provides a more ecologically valid representation of how emotional experiences tend to occur in daily life in the context of the laboratory.

If prior emotion and regulation effort are critical, it is also important to study neural activity underlying the emotion regulation to specific emotional stimuli as a function of individual differences in the everyday use of strategies to regulate emotion. Habitual use of cognitive reappraisal can influence neural responses to neutral stimuli preceded by emotional stimuli (Grupe et al., 2018). People who more regularly use cognitive reappraisal have less amygdala activation during reappraisal tasks (Fitzgerald et al., 2017), more fronto-cingulate activity during emotional conflict (Vanderhasselt et al., 2013), and more stress resilience, evidenced by biomarkers and self-report (Carlson et al., 2012).

Therefore, the second goal of the present study is to examine the role of the between-subject factor of habitual reappraisal in how participants respond to the experimental manipulation of emotion context. As indicated, we systematically vary the temporal sequencing of emotional affective qualities (i.e., neutral to negative emotional stimuli, and negative to neutral emotional stimuli). Emotion regulation can be accomplished by either conscious, explicit strategies such as cognitive re-appraisal, or automatic, implicit processes. Implicit, involuntary emotion regulation is not carried out following instructions, but simply evoked automatically without conscious monitoring of emotional state (Mauss et al., 2007). Habitual emotion regulation can reflect both explicit and implicit emotion regulation processes (Gyurak et al., 2011). Explicit and implicit regulation are not mutually exclusive, and emotion transitions are likely to evoke implicit emotion regulation processes even during deliberate cognitive reappraisal processes. We expect that individuals who frequently use reappraisal strategies will cope more effectively with dynamic emotional stimuli.

Implicit emotion regulation involves the engagement of the ventral anterior cingulate cortex (ACC) and the ventromedial PFC (vmPFC) in modulating subcortical activity; in contrast, explicit emotion regulation strategies, such as applying cognitive reappraisal, engages the prefrontal regions [e.g., ventrolateral PFC (vlPFC)] and the posterior parietal cortex (PPC) (Etkin et al., 2015). We utilized electroencephalography (EEG) source localization to identify functional connectivity (FC) involved in the implicit and explicit emotion regulation of the vmPFC-ACC and the vlPFC-PPC, respectively. With EEG, we can also assess functions in different frequency bands. Specifically, EEG beta band oscillation moderates long-distance cortical communication (Gross et al., 2004). Prefrontal-posterior coupling in the beta band has been shown in emotional processing (Miskovic and Schmidt, 2010) and emotion transitions (Hao et al., 2019). EEG alpha band oscillation relates to cognitive functions (Babiloni et al., 2006), such as inhibition and attentional demands (Ray and Cole, 1985). Furthermore, alpha synchronization has been shown to be related to working memory (Sauseng et al., 2005), which would be essential to processing temporal context information.

For the first research question, we hypothesized that FC would show different patterns in emotion transitions during a passive watch in comparison to a deliberate cognitive reappraisal; namely, a significant three-way interaction of emotional content, context, and regulation strategy. For the second research question, individuals who do not typically use cognitive reappraisal would be more influenced by context: (1) a constant neural response during neutral stimulus preceded by a negative emotion stimulus, and/or (2) the neural response fails to elevate when the second emotional stimulus is negative.

## Methods

### Participants

Thirty-six healthy right-handed college students participated in this study. Recruitment exclusion criteria were no history of mental disorders. Due to incomplete data or equipment malfunction, 32 participants remained for the final sample (female 66%, 21.5 ± 3.5 yo). Participants were requested to refrain from alcohol, caffeine, and other stimulants 4 h before the experiment. Written informed consent was obtained, and participation was compensated with course credit or US$20. This study was approved by the Institutional Review Board of Cornell University.

### Design

**Figure 1:**
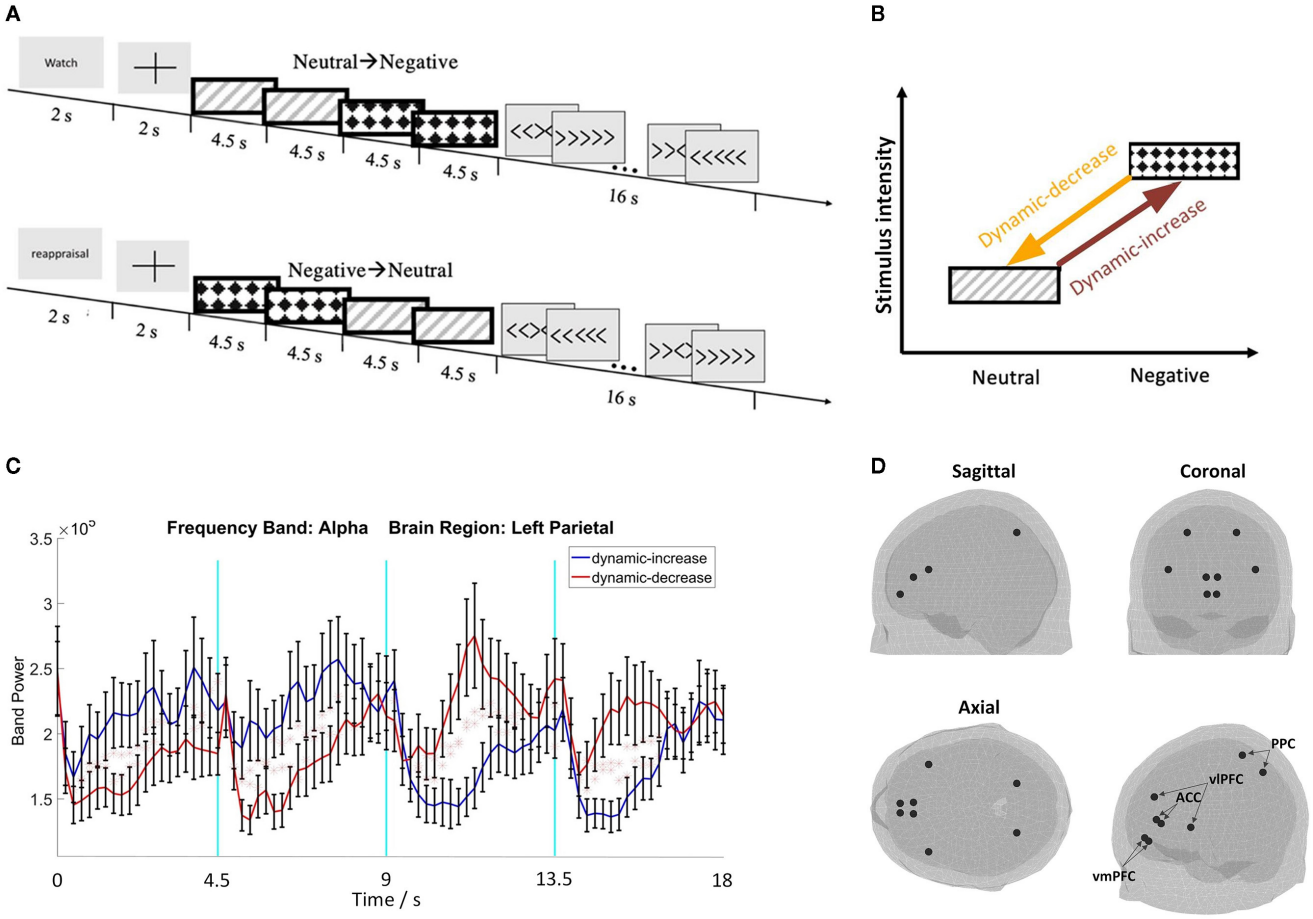
**(A)** Schematic representation of the two image sequence conditions with the watch or reappraisal tasks; **(B)** schematic plot of the emotion transition elicited by image sequences from low to high intensity (i.e., dynamic increase) or from high to low intensity (i.e., dynamic decrease); **(C)** representative electroencephalography (EEG) waveforms with standard errors (*N* = 32); **(D)** 3D brain maps of ROIs for source reconstruction.

Negative, emotionally threatening images (valence: *M* = 2.37, *SD* = 0.65, arousal: *M* = 5.95, *SD* = 0.77) and low arousal neutral images (valence: *M* = 5.12, *SD* = 0.53, arousal: *M* = 3.17, *SD* = 0.66) were selected from the IAPS database (Lang et al., 1997) (Supplementary material summarizes the image numbers and session information). Each image sequence comprised of 4 images, each lasting 4.5 seconds. Shown in Figures 1A,B, image sequences contained images either transitioning from neutral to negative as *dynamic-increase* condition, or from negative to neutral as *dynamic-decrease* condition.

Participants were instructed to either passively watch or cognitively reappraise each image sequence (18 trials in each task each condition). The words “watch” or “reappraisal” were shown on the screen for 2 seconds before each sequence. In the watch task, participants observed the stimulus image and experienced whatever feeling/emotion the image evoked without trying to change or alter their emotions. The reappraisal task required participants to re-interpret the meaning of the images so that their negative affect was decreased. Participants practiced cognitive reappraisal before the formal session and were assisted if they used other emotion regulation strategies, such as distracting themselves from the negative stimuli or suppressing their emotions. This experimental manipulation reliably induced different neural responses between the watch and reappraisal tasks (e.g., Thiruchselvam et al., 2011; Kim et al., 2013). To further validate the reappraisal manipulation, heart-rate variability (HRV) was assessed to indicate regulated emotional responding (Thayer et al., 2012). After each image sequence, a Flanker task was followed as a distraction task to eliminate residual emotional influence.

### Measures

We administered the six-item Emotion Regulation Questionnaire to assess individual differences in cognitive reappraisal (Gross and John, 2003a). Sample questions included: “When I want to feel less *negative* emotion (such as sadness or anger), I *change what I'm thinking about*,” or “When I want to feel more *positive* emotion (such as joy or amusement), I *change what I'm thinking about*.” It is a well-established instrument with strong psychometric properties that has been used across a wide range of samples (Spaapen et al., 2014).

A 128-channel BioSemi EEG device (BioSemi B.V., Amsterdam) was used to record EEG at a 512 Hz sampling rate. We collected electrocardiograph data from the three BioSemi electrodes and estimated HRV using the Poincare plot (Brennan et al., 2002). In the EEG preprocessing procedure, all EEG channels were referenced to the algebraic average of left and right mastoids. Then, EEG signals were bandpass filtered between 1 and 40 Hz, using a two-way least-squares finite impulse response filter in EEGLab (Delorme and Makeig, 2004). Bad channels were visually identified and spherically interpolated. Next, the data were epoched by each image. Epochs with obvious abnormal signal segments were excluded for Independent Component Analysis (ICA). Then, the extended Infomax ICA (Bell and Sejnowski, 1995), which was implemented in EEGlab as the default ICA algorithm, was utilized to detect and remove artifacts contaminated by eye movements, muscle, and cardiac artifacts. We determined the ICA components by the ICA maps and also the power spectrum of the ICA component (Delorme and Makeig, 2004). After removing the artifact components, the ICA source signals were transferred back to the original signal space, which was then used for the subsequent analysis.

The center locations of the vlPFC, vmPFC, ACC, and PPC regions were used to extract the source signal (the size of the region was determined by the atlas profile). The location was determined by using the FieldTrip toolbox and the “ROI_MNI_V4.nii” atlas profile, from which the area of the selected regions can be derived (Oostenveld et al., 2011; Evans et al., 2012). Figures 1C,D show representative EEG waveforms and the brain maps of region of interests (ROIs).

The brain was divided into regular three-dimensional grids, and the source strength for each grid point was computed. The method applied in this study is termed Linearly Constrained Minimum Variance (LCMV), and the estimates were calculated in the time domain (Van Veen et al., 1997). The beamformer estimated brain activity separately for each source location. For each location, a beamformer found a spatial projection of the observed signal, such that signals from that location were preserved, while contributions from all other signals were maximally suppressed. The LCMV beamformer did this by minimizing the variance of the filtered signal subject to a unit-gain constraint (that is, the product of filter and forward matrix at the desired location is enforced to be the identity matrix).

The EEG signal was segmented into 2-second epochs, with 1-second overlap in the entire images excluding the fixation period. Each segment was multiplied with a hamming window before the calculation of the Fourier transform. We denoted the k-*th* segment of the i-*th* time series by *x*_*i,k*_(*t*), and its Fourier transform by *X*_*i,k*_(*f*). The cross-spectral is defined as

$$\begin{array}{l} S_{i,k}\left(f\right)=\frac{1}{K}\overset{K}{\underset{k=1}{\sum }}X_{i,k}\left(f\right){X_{j,k}\left(f\right)}^{*} \end{array}$$

where (·)^*^ denotes complex conjugation.

The coherence between *x*_*i*_(*t*) and *x*_*j*_(*t*) is defined as

$$\begin{array}{l} C_{i,j}\left(f\right)=\frac{S_{i,j}\left(f\right)}{\sqrt{S_{i,i}\left(f\right)S_{j,j}\left(f\right)}} \end{array}$$

The imaginary coherence is defined as

$$\begin{array}{l} iCoh_{i,j}\left(f\right)=|\varsigma \left(C_{i,j}\left(f\right)\right)| \end{array}$$

where *st* denotes the operator that takes the imagery part of a complex value and |.| denotes the absolute operation.

The theoretical robustness obtained for the method is based on the imaginary part of coherence (Nolte et al., 2004, 2008). The robustness is defined as the property of a measure to yield asymptotically zero connectivity for linear mixtures of independent signals (Mahjoory et al., 2017). We limited our analyses to robust connectivity metrics in order to avoid basing our results on spurious connectivity, which might be caused by volume conductivity (Bastos and Schoffelen, 2016).

The FC between vmPFC and ACC, denoted as FC (vmPFC-ACC), and the FC between vlPFC and PPC, denoted as FC (vlPFC-PPC), in the alpha band (8–13 Hz) and the beta band (14–30 Hz) were calculated. For each person, we computed the average coherence of the images with the same emotional category (the first and last images) in each condition under each task.

### Analysis

We validated the reappraisal task manipulation with the HRV measure by conducting a mixed-effects model where each participant was designated as a random effect and the regulation task was as a within-subject factor. We first tested the three-way interaction of content (neutral vs. negative), context (dynamic-increase vs. dynamic-decrease), and regulation task (watch vs. reappraisal) on FC (vmPFC-ACC) and FC (vlPFC-PPC) in the alpha and beta band, respectively. Linear mixed models were conducted with all three factors as within-subject factors and each participant as a random effect.

To assess the between-subject factor in emotion transition, a three-way interaction of content, context, and habitual reappraisal was tested separately on watch and reappraisal task. Mixed models, including content and task as within-subject factors and habitual reappraisal (continuous variable, *M* = 29.34, *SD* = 5.92) as a between-subject factor, were conducted on the above-mentioned outcome variables.

## Results

### Neural Responses to Emotion Transitions and Emotion Regulation

The reappraisal task evoked higher HRV than the watch task across image conditions, χ^2^ (1) = 5.695, *p* = 0.017, which provides further validation of our task protocol because elevated HRV indicates more effective coping (Denson et al., 2011; Thayer et al., 2012).

**Figure 2:**
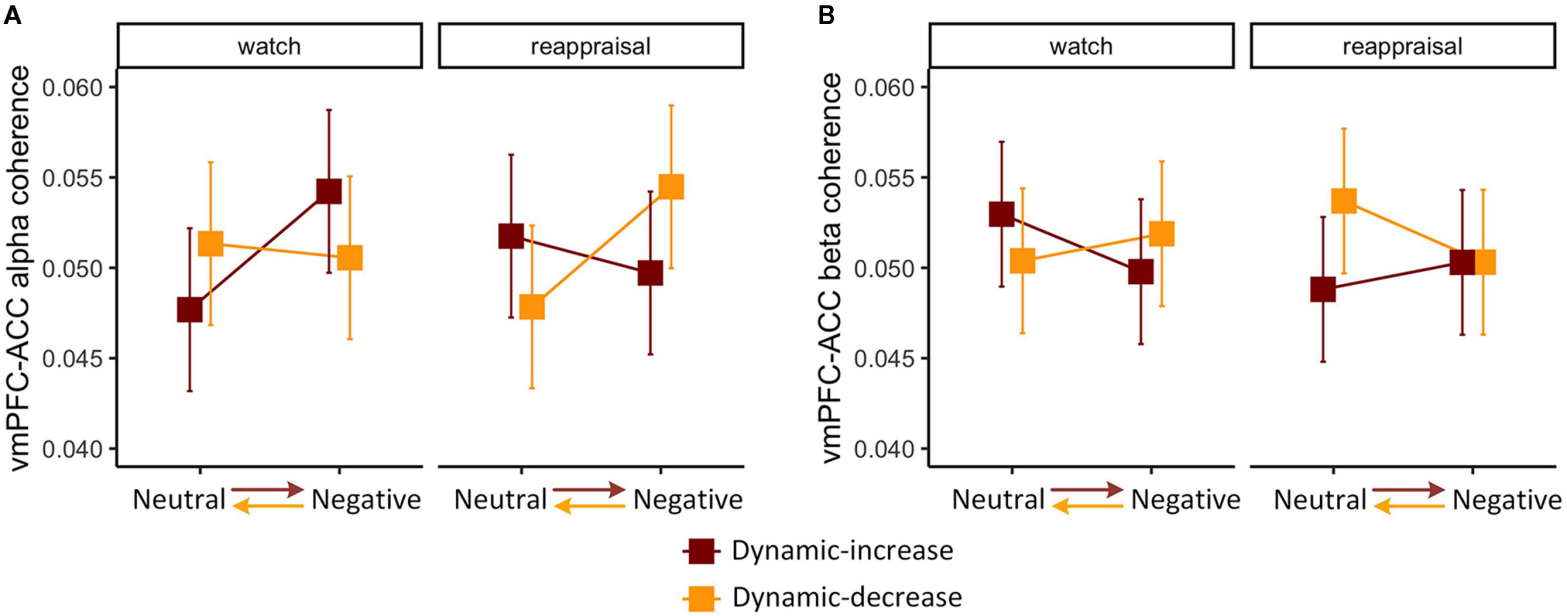
Three-way interaction plot of model estimates of content, context, and task on the **(A)** alpha band FC between the ventromedial prefrontal cortex (vmPFC) and the anterior cingulate cortex (ACC) and **(B)** beta band FC between vmPFC and ACC. Error bars are standard errors.

The three-way interaction of emotional stimuli content, temporal context, and regulation task was significant on FC (vmPFC-ACC) in alpha band, χ^2^ (1) = 5.213, *p* = 0.022 (Figure 2A). The three-way interaction was also significant on FC (vmPFC-ACC) in beta band, χ^2^ (1) = 4.990, *p* = 0.026 (Figure 2B), which manifested the opposite interaction pattern of the alpha band. This means that the interaction of emotion content and regulation on coherence varied by context. When images shifted affective intensity, FC (vmPFC-ACC) in the alpha band increased in the watch task and decreased in the reappraisal task, whereas FC in beta band manifested a reciprocal pattern.

**Figure 3:**
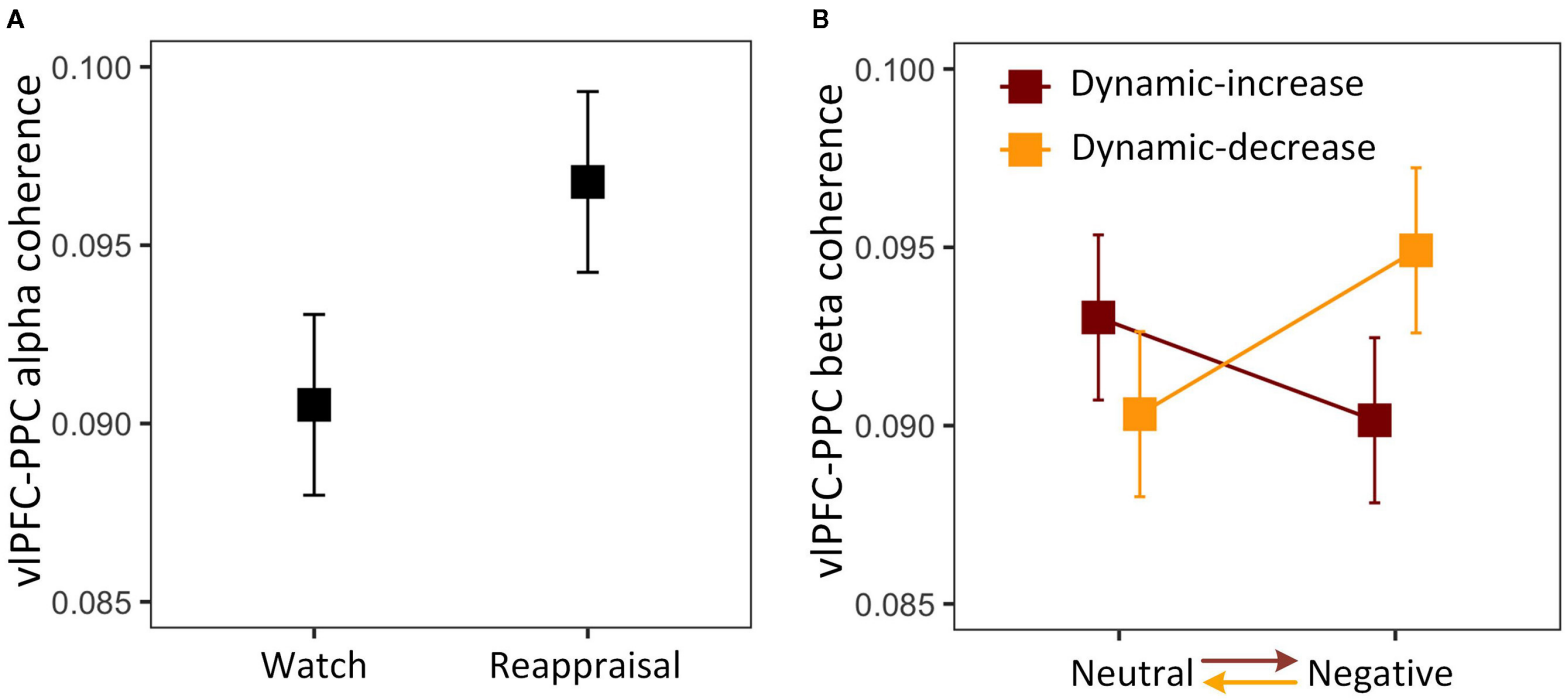
**(A)** Main effect of reappraisal task on the alpha band FC between the ventrolateral prefrontal cortex (vlPFC) and the posterior parietal cortex (PPC); **(B)** two-way interaction plot of model estimates of content and context on the beta band FC between vlPFC and PPC. Error bars are standard errors.

The three-way interaction on FC (vlPFC-PPC) in the alpha band was not significant, χ^2^ (1) = 2.076, *p* = 0.153. The analysis was then reduced to a one-way model, showing a significant task main effect, χ^2^ (1) = 3.942, *p* = 0.047 (Figure 3A). The coupling between vlPFC and PPC was stronger in the reappraisal task, which implies that the reappraisal recruited the frontoparietal network supporting cognitive control. The three-way interaction was also not significant in FC (vlPFC-PPC) in the beta band, χ^2^ (1) = 0.431, *p* = 0.511. Thus, the model was reduced to a two-way interaction of content and context, χ^2^ (1) = 4.289, *p* = 0.038, showing that the effect of emotional content on coherence depended on context (Figure 3B). Negative stimulus presented at the beginning evoked stronger connectivity than if it was presented later. It also shows a reduction in prefrontal-posterior coupling in beta band regardless of the direction of transitions and strategies.

### Habitual Reappraisal in Relation to Neural Responses to Emotion Transitions

**Figure 4:**
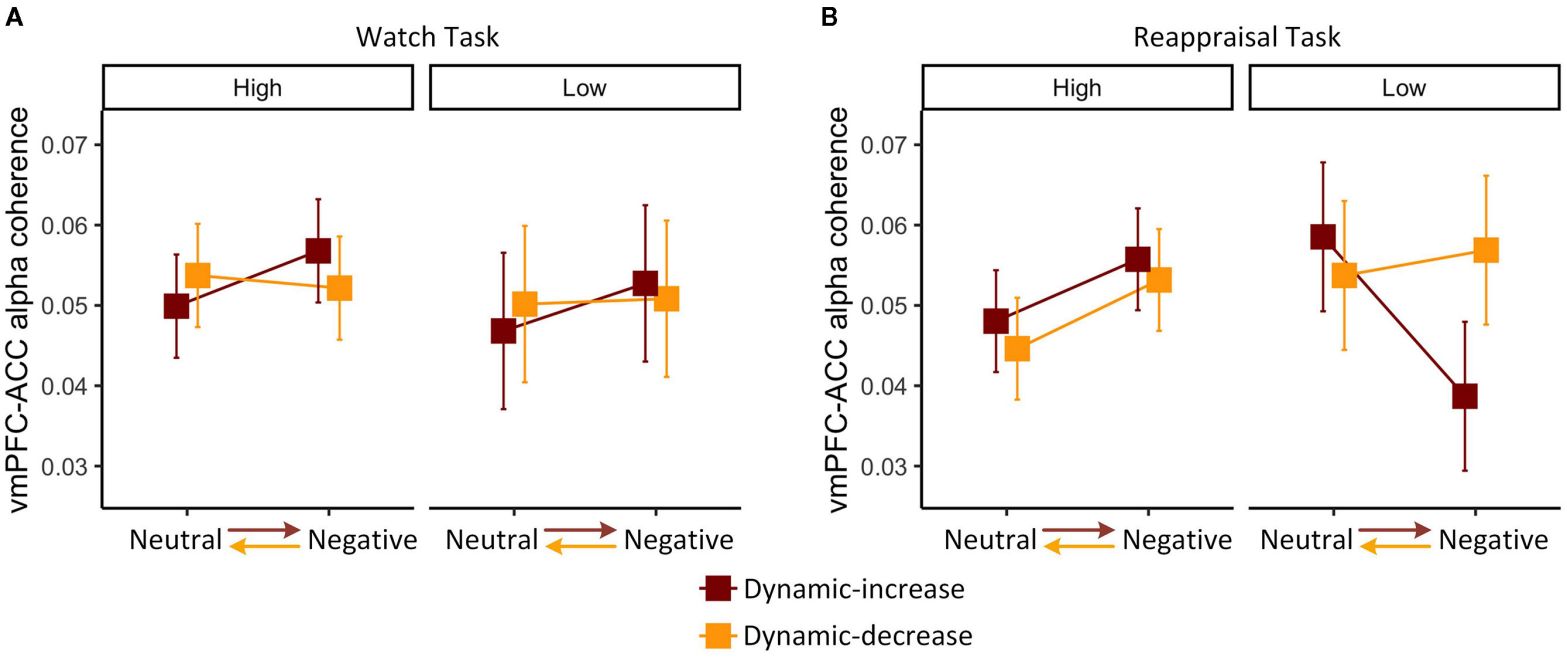
Three-way interaction plot of model estimates of content, context, and habitual reappraisal (high vs. low: mean ± 1 *SD*) on the alpha band FC between vmPFC and ACC in the **(A)** watch task and **(B)** reappraisal task. Error bars are standard errors.

We then examined whether individual differences in habitual cognitive reappraisal based upon the Emotional Regulation Questionnaire would moderate the findings above. For the watch task, habitual reappraisal did not moderate dynamic emotion transitions on FC (vmPFC-ACC) in the alpha band, χ^2^ *(1)* = 0.044, *p* = 0.834 (Figure 4A). For the reappraisal task, the three-way interaction of content, context, and habitual reappraisal was significant, χ^2^ (1) = 3.920, *p* = 0.048 (Figure 4B). *Post-hoc* comparison of the reappraisal trend showed that habitual reappraisal was highly correlated with FC (vmPFC-ACC) in the alpha band in the dynamic-increase condition (*p* = 0.005, *R*^2^ = 0.270), but not in the dynamic-decrease condition (*p* = 0.903, *R*^2^ = 0.020). These results suggest that when people applied cognitive reappraisal strategy, individual differences in habitual reappraisal were manifested in the FC (vmPFC-ACC) in alpha band. In addition, habitual reappraisal did not moderate the FC (vmPFC-ACC) in beta band and the FC (vlPFC-PPC) in both bands.

Together with Figure 2A, it can be inferred that the interaction of content and context during watch task on the FC (vmPFC-ACC) in alpha band did not vary across participants. However, during the reappraisal task, FC slightly decreased in dynamic-increase condition, mainly because people with less habitual reappraisal dramatically decreased their FC (vmPFC-ACC) in alpha band.

## Discussion

The present study investigated the EEG FC during an emotion regulation task. Unlike most research on emotion regulation, we were particularly interested in the situation where more than one emotional category occurs in close proximity. This is potentially important for two reasons. First, as we argued in the introduction, there are reasons to suspect that emotional context and timing matters for the neural responses of emotional experiences. Second, in real life, we rarely experience a single emotion only, devoid of contextual factors such as other emotional experiences. We first demonstrated that FC between frontal regions (vmPFC and ACC) differed depending on the interaction of three factors: whether or not the participant was engaged in emotion regulation (i.e., cognitive reappraisal), the affective content of the stimulus (i.e., negative vs. neutral), and whether or not the stimulus had been preceded by another stimulus of differing emotional content (i.e., context). We also demonstrated that prefrontal-posterior coupling (vlPFC and PPC) depended on these same three factors, albeit in a different manner. In addition to task emotion regulation, we also investigated personal emotion regulation traits. We found that participants who less frequently used cognitive reappraisal were more influenced by temporal context; specifically, the neural response failed to elevate when the second emotional stimulus was negative.

### Emotion Regulation in Emotion Transitions

Our study suggests that, when the images shifted affective intensity, FC (vmPFC-ACC) in the alpha band increased in the watch task and decreased in the reappraisal task, whereas FC in the beta band manifested a reciprocal trend. Given the involvement of PFC-ACC in implicit emotional regulation (Etkin et al., 2015), this pattern of alpha FC may reflect participants engaging more conflict processing underlying the implicit emotion regulation during emotion transitions. Recovery from the previous stimulus response and reactivity to the new stimulus might interrelate with conflict generated and resolved by regulation (Etkin et al., 2006, 2010). Conflict could induce negative affect reflected in ACC activation (Braem et al., 2017). Furthermore, the ACC is related to subsequent memory retrieval (Medford et al., 2005). Also, medial PFC is activated if the emotional experience is sustained after a stimulus terminates (Waugh et al., 2014, 2017), and integrates contextual and memorial information to update affective sensory-perceptual information (Roy et al., 2012; Delgado et al., 2016). Moreover, Résibois et al. (2017) showed that these two regions coordinate during dynamic emotion responses with medial PFC, particularly relating to emotion onset and ACC being responsible for emotion offset.

In addition, FC (vlPFC-PPC) in beta band again demonstrated temporal context effect, showing that the prefrontal-posterior coupling decreased during emotion transitions regardless of emotional content. This replicates our previous findings (Hao et al., 2019) and extends prior results with passive watch to deliberate emotion regulation. The PPC underlies perceptual input processing (Etkin et al., 2015; Silvers and Moreira, 2019) and lateral PFC may reflect a general inhibitory mechanism responsible for limiting the impact that an emotional state from one event has on the emotional state of a subsequent event (Waugh et al., 2017).

In the reappraisal task, FC (vlPFC-PPC) in the alpha band was significantly higher when participants were explicitly regulating their emotions than when they were not, regardless of context conditions. The predominance of right hemispheric frontoparietal network-enabled cognitive control facilitates emotion regulation (Hendricks and Buchanan, 2016). This provides further indirect evidence for the validity of our reappraisal task manipulation.

### Habitual Reappraisal Moderates Regulating Emotion Transitions

Emotional events could trigger emotion transitions wherein neural mechanisms operate as a function of emotion regulation effort *in situ* and in relation to more general individual differences in emotion regulation. During the process of emotional regulation, for individuals high in habitual reappraisal, FC (vmPFC-ACC) in the alpha band was similar regardless of stimulus transition, whereas for non-habitual users, FC differed depending on both content and context: low connectivity for negative content stimulus preceded by a neutral stimulus, and higher connectivity for the other conditions. This pattern of findings suggests that when individuals who tend not to use cognitive reappraisal of emotions do engage in emotional regulation, they are more affected by the change in affective emotional content than habitual users are. When participants were not engaged in emotional regulation, users and non-users showed the same patterns of frontal connectivity.

Individuals with higher habitual reappraisal showed more flexible neural responses to shifting affective stimuli per their current content with less dependence on the prior emotion stimulus. Participants may employ both implicit and explicit emotion regulation processes together when regulating responses to varying emotional stimuli. Individuals who more frequently use reappraisal may be able to recruit comparable implicit emotion regulation related neural circuits in conjunction with recruiting explicit emotion regulation related neural circuits during emotion transitions. Reappraisal frequency and working memory capacity are positively associated with reappraisal ability (McRae et al., 2012). Individuals who frequently use cognitive reappraisal in their daily lives are more likely to spend fewer cognitive resources during emotion regulation and require fewer cortical resources to achieve the same reduction in activation of the ventral emotion generation areas, thus showing greater neural efficiency (Drabant et al., 2009; Ortner et al., 2016). Such efficiency could be related to flexible neural responses and more automatic regulation processes during emotion transitions.

An alternative explanation is that people may time their regulation when emotional stimuli are experienced sequentially. Emotion regulation can be engaged at various points as an emotional episode unfolds over time. The success of emotion regulation depends on regulation timing (Urry, 2009). The earlier the emotion-regulatory process occurs, the lesser effort with regulation efficacy which is relatively unaffected by the level of emotional intensity. By contrast, regulation strategies that occur at the later emotion processing stages require effort proportional to the intensity of the emotional response (Sheppes and Gross, 2011). Correspondingly, the timing of regulation in an emotional sequence with varying intensity may influence the outcome. Individuals high in habitual reappraisal might start to engage in reappraisal early regardless of the perceived intensity of the current stimuli in an image, which well-prepares them to engage in flexible control effectively.

### Limitations and Future Development

This study provides preliminary insight into the temporal unfolding of affective responses when regulating emotion transitions in the brain. This may have potential clinical implications for understanding individual vulnerability to environmental fluctuations. Individuals who are flexible tend to be more resilient. Future studies that take complex social contexts into account will permit a more complete analysis of emotion regulation during emotion transitions. These can then be related to individual differences (e.g., anxiety) associated with environmental adaptation or adverse psychological health.

A second contribution is our demonstration that electrophysiology can provide frequency domain information regarding affective and cognitive functions. Our data afforded the possibility that alpha and beta frequency bands may play different roles in emotion transitions, which awaits further studies with formalized hypotheses to test.

Although we validated the cognitive reappraisal task with HRV, assessing other physiological variables (e.g., corrugator and zygomatic facial electromyography and skin conductance response) would provide additional evidence. Although we can be confident that the transition between neutral and negative affect images has reliable neurodynamic consequences, we are unable to comment on the effects of emotion transitions in general. Additional affect transitions (e.g., negative to positive and the obverse) with different presentation timing would be valuable to gauge the generalizability of our results. In the future, we also plan to test the Gamma band coherence in individual differences of emotion transitions, as it has been shown to be related to emotion detection (Li and Lu, 2009; Yang et al., 2020). It would also be valuable to examine our research questions with functional MRI (fMRI) in the future given its better spatial resolution than EEG in capturing specific brain activation; although the faster and more precise temporal aspects of EEG are critical, particularly when analyzing neurocognitive responses to changing emotions in real-time.

In sum, emotion regulation during emotion transitions may reflect individual differences in neural activities. Since adaptive emotion regulation requires flexibility (Aldao et al., 2015), in a broader sense, the present study suggests that flexible responses to emotions are likely to contribute to well-being. Individuals more experienced in cognitive reappraisal of emotions in daily life appear better able to flexibly recruit neural activities to respond to the dynamic, ever-changing emotional experiences encountered in daily life.

## Data Availability Statement

The raw data supporting the conclusions of this article will be made available by the authors, without undue reservation.

## Ethics Statement

The studies involving human participants were reviewed and approved by Cornell University. The patients/participants provided their written informed consent to participate in this study.

## Author Contributions

YH designed the research program, collected the data, and drafted the manuscript. YH and LY performed the data analysis. GE supervised the research and provided critical feedback on drafts. All authors contributed to the article and approved the submitted version.

## Conflict of Interest

The authors declare that the research was conducted in the absence of any commercial or financial relationships that could be construed as a potential conflict of interest.

## Publisher's Note

All claims expressed in this article are solely those of the authors and do not necessarily represent those of their affiliated organizations, or those of the publisher, the editors and the reviewers. Any product that may be evaluated in this article, or claim that may be made by its manufacturer, is not guaranteed or endorsed by the publisher.
